# Comparative Evaluation of Additives in Softwood Fractionation: Impacts on Lignin Recovery and Pulp Quality

**DOI:** 10.1002/cssc.70640

**Published:** 2026-04-18

**Authors:** Juho Antti Sirviö, Ekaterina Sheridan, Donya Arjmandi, Jasmiina Haverinen, Dmitry Tarasov, Chunlin Xu, Ari Ämmälä, Jarkko Räty

**Affiliations:** ^1^ Fibre and Particle Engineering Research Unit University of Oulu Oulu Finland; ^2^ Max Planck Institut für Kolloid‐ und Grenzflächenforschung Am Mühlenberg 1 Potsdam Germany; ^3^ Laboratory of Natural Materials Technology Åbo Akademi University Turku Finland; ^4^ Kajaani University Consortium Measurement Technology Unit University of Oulu Kajaani Finland

**Keywords:** biomass, cellulose, fractionation, green chemistry, lignin

## Abstract

Lignocellulosic biomass fractionation with concurrent lignin stabilization via chemical modification has been shown to enhance delignification efficiency and enable the recovery of lignin with a low degree of condensation, thereby increasing the application potential of both carbohydrate and lignin fractions. In this study, five different chemicals—ethylene glycol (EG), glyoxylic acid (GlyoxA), phenol (Phen), thiourea (ThioU), and thiolactic acid (TLA)—were evaluated for their ability to modify lignin during acid hydrotropic fractionation (AHF) of softwood in aqueous *p*‐toluenesulfonic acid. Their effects were compared to AHF without additives. Among the tested modifiers, Phen achieved the highest lignin removal. However, TLA led to the highest recovery lignin yield, the lightest‐colored fractions, and pulp sheets with superior tensile properties, highlighting the strong potential of thiol‐based nucleophiles in wood delignification. ThioU, another sulfur‐based nucleophile, also produced lightly colored fractions, but lignin redeposition on fibers negatively impacted pulp mechanical properties. GlyoxA moderately improved lignin removal and yield, while EG had negligible effect compared to plain AHF. Notably, well‐defined, spherical lignin nanoparticles were obtained from TLA‐ and ThioU‐modified lignins, although ThioU‐lignin also formed film‐like structures due to nonprecipitated lignin. Other lignin samples yielded irregularly shaped nanoparticles.

## Graphical abstract

Chemical lignin stabilization during acid hydrotropic fractionation is shown to distinctly influence delignification, lignin quality, and pulp performance. Phenol enhances lignin removal, while thiol‐based modifier yields light‐colored lignin, high recovery, and strong pulp. Thiol modifier also generates well‐defined lignin nanoparticles, revealing their strong potential for tailoring both lignin structure and material functionality.

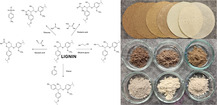

## 1 Introduction

Fractionation of lignocellulosic biomass is a cornerstone of biorefinery processes. Traditionally, the primary focus has been on carbohydrate fractions, particularly cellulose, which is extensively utilized in the paper and packaging industries through pulping [1]. During pulping, lignin and a portion of hemicelluloses are solubilized, with cellulosic fibers serving as the main product. Although lignin can potentially be recovered from the washing liquors, it—along with dissolved carbohydrates—is typically combusted to regenerate process chemicals and meet the substantial energy demands of conventional pulping operations [2].

Growing awareness of the environmental drawbacks associated with fossil‐based materials and chemicals has intensified interest in biobased alternatives, driving demand for novel fractionation methods [3, 4, 5, 6]. These methods aim to isolate lignocellulosic components in an environmentally benign manner—characterized by low energy consumption and the use of nonhazardous chemicals. Particular emphasis has been placed on the recovery of high‐quality lignin, leading to the emergence of the “lignin‐first” biorefinery concept [7]. As the most abundant natural source of aromatic compounds, lignin holds significant promise as a biobased substitute for petroleum‐derived products across various applications [8, 9, 10]. Simultaneously, the rising demand for sustainable packaging materials, such as fiber‐based cardboard [11], underscores the need for fractionation strategies capable of producing both high‐quality lignin and carbohydrate fractions.

One of the major drawbacks of traditional pulping processes, such as the kraft method, is their high energy consumption, which stems from elevated temperatures, pressures, and prolonged reaction times. These harsh conditions also lead to significant chemical alterations in lignocellulosic components—most notably, lignin condensation [12, 13] and irreversible aggregation of microfibrils, commonly referred to as hornification of the fiber cell wall [14]. Lignin condensation reduces its reactivity [13] and contributes to the darkening of the resulting cellulosic pulp due to residual lignin [1]. Additionally, the downstream valorization of condensed lignin into platform chemicals is hindered by the formation of strong carbon–carbon (C—C) bonds [15].

As increasing emphasis is placed on both lignin isolation and delignification efficiency, the use of chemicals that specifically target lignin structure has gained attention [16]. Depending on the intended outcome, these additives can either protect/stabilize or chemically modify lignin. Protective agents can react with lignin during fractionation and be removed afterward. A notable example is aldehyde‐assisted fractionation, which employs detachable protecting groups [17]. Aldehydes react with lignin diol structures—particularly those in β‐O‐4 linkages—forming acetals that prevent lignin fragmentation and the formation of highly reactive carbocations, a key driver of lignin condensation during isolation. Alcohols, such as diols, can also form ether bonds with lignin [18], which are more hydrolytically stable than native hydroxy groups [19]. Alternatively, selective lignin condensation can be exploited by introducing monomeric phenolics, which form C—C bonds with lignin fragments similar to those formed during self‐condensation [20, 21, 22, 23, 24]. This approach only mildly increases lignin conjugation and can yield lignin with a lighter color [25, 26]. Furthermore, phenol‐protected lignin can be further valorized, for example, through hydrogenation into biobased bisphenol building blocks [20].

Strong nucleophilic compounds, such as thiols, represent promising candidates for lignin modification during delignification and fractionation processes. For instance, cysteine has been employed in lignin isolation from various biomass sources [27, 28, 29]. Moreover, thiolactic acid (TLA) has demonstrated the ability to produce high‐yield, white lignin from softwood [30]. Although the exact reaction mechanism of thiol‐based fractionation remains incompletely characterized, it is presumed to follow the principles of thioacidolysis—a well‐established analytical method for lignin structure elucidation [31]. Similar to thiols, thiourea (ThioU) can act as a carbocation scavenger, forming chemically modified lignin with isothiouronium groups that can be hydrolyzed to thiols [32].

**FIGURE 1:**
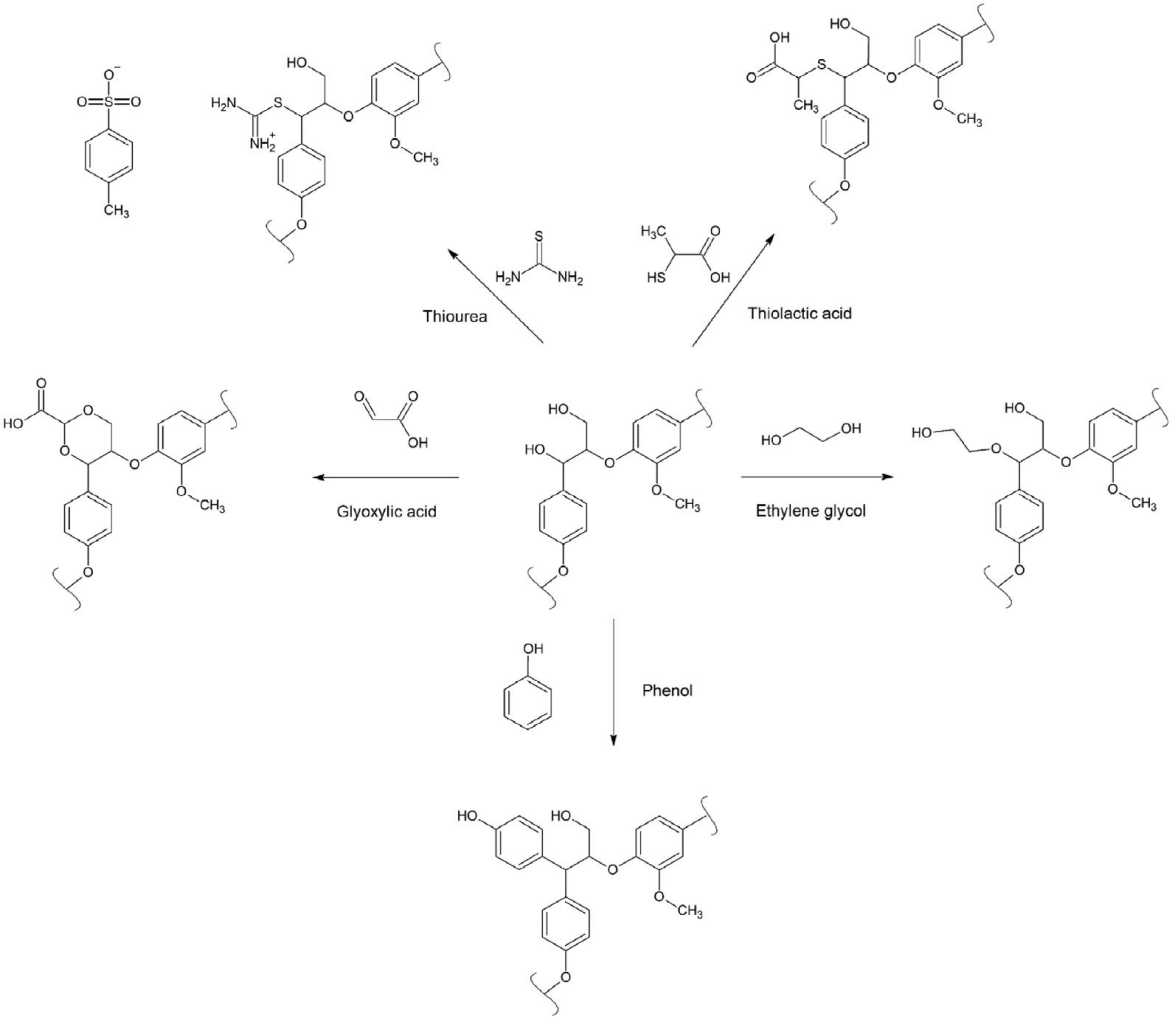
Chemicals studied and the corresponding chemical structure of β‐O‐4 linkage after chemical modification via selected chemicals.

Although numerous studies have explored the effects of additives on biomass fractionation, direct comparisons between different lignin modification strategies—particularly using the same biomass species—remain scarce. Comparative studies could provide valuable insights for designing more efficient lignocellulosic biomass fractionation methods. In this study, five additives—ethylene glycol (EG), glyoxylic acid (GA), phenol (Phen), ThioU, and TLA—were investigated as modifying agents during AHF of softwood sawdust. The chemical structures of the additives and the corresponding modified β‐O‐4 lignin linkages are illustrated in Figure 1. While the reactions of EG, GA, and Phen with lignin are well‐documented, the mechanisms involving ThioU and TLA are less understood, and mechanism presented here should be considered as tentative.

AFH has been shown to efficiently dissolve lignin from nonwood and hardwood biomass under mild conditions [6, 33]. However, significantly lower delignification efficiency has been reported for softwood [34]. Therefore, softwood serves as a suitable reference material for evaluating whether the introduction of additional chemicals can enhance lignin removal. In this study, the effects of five additives on lignin and carbohydrate content in the solid fiber fraction were investigated and compared to plain AHF without additives. The yield and properties of the isolated lignin were also assessed. Additionally, the mechanical properties of fiber sheets produced from the solid fraction were characterized, and the potential for producing nanolignin from the different lignin fractions was explored.

## 2 Materials and Methods

### 2.1 Materials

Spruce sawdust used as a raw material were kindly provided by Keitele Forest Oy at solid content of 50 wt%. Sawdust was stored in fridge at —18°C and thawed prior using. TLA (≥97.0%), was obtained from TCI (Germany), and p‐toluenesulfonic acid monohydrate (≥98%), ThioU (≥99.0%), GA (98%), and GA (as monohydrate, 98%) from Sigma–Aldrich (Germany), EG (95%) from Fisher Science, Phen (99+%) and ethanol (96%) from VWR (United States), and uranyl acetate from Polysciences. Deionized water was used for all the experiments.

### 2.2 Delignification of Softwood

Plain AHF (Ref), without the addition of lignin‐modifying agents, was used as the reference method and conducted according to a previously reported procedure with minor modifications [25]. To prepare the AHF solution, 80 g of *p*‐toluenesulfonic acid monohydrate (final *p*‐toluenesulfonic acid concentration 72 wt%) was dissolved in water to obtain 100 g of AHF mixture. The solution was heated in an oil bath at 80°C with magnetic stirring until a clear liquid was formed. Subsequently, 20 g of nondried spruce sawdust (equivalent to 10 g dry solids) was added and reacted under continuous stirring for 30 min.

After the reaction, the mixture was diluted with 100 mL of ethanol and filtered. The solid cellulosic fraction (i.e., the undissolved portion of the wood) was dispersed in 200 mL of ethanol, followed by filtration and washing with 50 mL of ethanol. The ethanolic washing liquors were collected, and the solid fraction was further washed with 500 mL of deionized water. The final product was stored in a nondried state at 4°C.

The baseline dosage for lignin‐modifying chemicals was adapted from a previous study [35], where 6 wt% of GA, corresponding to 8.1 mmol per gram of wood, was used. This molar ratio was applied to all additives in this study. AHF with lignin‐modifying agents was performed similarly to the plain AHF, except that the calculated amount of water was replaced with the respective chemical. The compositions of the reaction mixtures are presented in Table S1.

Lignin was precipitated from the ethanolic washing liquor by adding a threefold excess volume of water. The precipitated lignin was filtered and washed with 200 mL of water and then redispersed in approximately 10 mL of ethanol. A second precipitation was performed by adding a threefold excess volume of water—except for ThioU‐lignin, where ethyl acetate was used instead. Ethyl acetate was used due to the high solubility of ThioU‐lignin in aqueous solvent at this step. The lignin was then filtered and washed with water (or ethyl acetate in the case of ThioU), collected, and dried in an oven at 60°C.

Lignin yield was calculated based on the original mass of the wood and referenced to the calculated lignin content in the original material. Lignin recovery was determined from the amount of dissolved lignin, defined as the difference between the lignin content of the original wood and the residual lignin remaining in the cellulosic fraction.

To obtain the factual lignin yield, the mass contributions of chemical groups introduced during the modification were subtracted from the measured lignin mass. For Phen‐lignin, the amount of incorporated phenolic groups was calculated based on the p‐hydroxyphenyl content. For GA‐ and TLA‐lignin, the amounts of introduced groups were quantified using the carboxylic acid content determined by ^31^P nuclear magnetic resonance (NMR). The factual yield of ThioU‐lignin was calculated from the amount of isothiouronium tosylate groups determined by elemental analysis.

### 2.3 Characterization of the Wood Before and After Delignification

The chemical composition of the original and AHF‐treated wood samples—including cellulose, hemicellulose, and lignin content—was determined using the National Renewable Energy Laboratory (NREL) standard protocol [36]. Prior to analysis, the samples were ground using a coffee grinder. The degree of polymerization (DP) of cellulose was measured using the cupriethylenediamine (CED) method in accordance with ISO 5351.

### 2.4 Preparation and Characterization of Cellulosic Sheet

The nondried cellulosic fraction was diluted to 0.3 wt% (corresponding to 0.5 g dry matter) and mixed for 1 min using an Ultra‐Turrax mixer at 12,000 rpm. The resulting dispersion was filtered through a polyvinylidene fluoride (PVDF) membrane with a pore size of 0.65 µm. After removing visible water, the moist sheet was covered with another PVDF membrane and dried between two paper sheets for 9 min at 93°C under vacuum (approximately 70 mbar).

Color values and ISO brightness (ISO 2470‐1) of the cellulosic sheets were measured using a Konica Minolta CM‐2600d spectrophotometer (Japan). Measurements were taken at three random positions per sheet, and results were reported as averages. The L* value represents lightness (0 = black, 100 = white), a* indicates the green–red axis (negative = green, positive = red), and b* reflects the blue–yellow axis (negative = blue, positive =  yellow).

Mechanical properties of the sheets were evaluated via tensile testing using a universal testing machine (Zwick D0724587, Switzerland) equipped with a 100 N load cell. Prior to testing, sheets were conditioned at 23°C and 50% relative humidity for 48 h and then cut into 5‐mm‐wide strips. Thickness was measured at three locations using a precision thickness gauge (Hanatek FT3, United Kingdom), and the average value was used. Tensile tests were conducted with a 40 mm gauge length and a strain rate of 4 mm/min. Five strips were tested per sample, and results were reported as averages.

The morphology of the cellulosic sheets was examined using optical and electron microscopy. Optical images were acquired with a Leica MZFLIII (Germany) microscope. For electron microscopy, a field emission scanning electron microscope (FESEM, JEOL JSM‐7900F, Japan) operating at 0.5 kV was used. Prior to FESEM analysis, samples were sputter‐coated with platinum.

### 2.5 Characterization of Lignin

The color values and ISO brightness (ISO 2470‐1) of the lignin were measured as described for fiber sheets. Ultraviolet–visible (UV–Vis) spectroscopy was used to assess the UV–Vis absorption properties of lignin solutions. Samples were dissolved in dimethyl sulfoxide (DMSO) at a concentration of 0.1 wt%, and spectra were recorded using a VWR UV‐6300 PC (China) UV–Vis spectrophotometer.

The chemical structure of lignin was analyzed using NMR spectroscopy. ^1^H and 2D heteronuclear single quantum coherence (HSQC) NMR spectra were acquired by dissolving 90 mg of lignin in 0.6 mL of DMSO‐d_6_. Measurements were performed in 5 mm NMR tubes using a Bruker AVANCE III 500 MHz (Germany) spectrometer equipped with a 5 mm Z‐gradient BBO (Broadband Observe) probe.

Methoxy (–OCH_3_) content was quantified via ^1^H NMR using 4‐nitrobenzaldehyde as an internal standard. Briefly, 15 mg of lignin was mixed with 5 mg of 4‐nitrobenzaldehyde and dissolved in 0.6 mL of DMSO‐d_6_. The mixture was transferred to a 5 mm NMR tube and analyzed using the same spectrometer.

Hydroxy and carboxylic acid group contents were determined using quantitative ^31^P NMR spectroscopy. For this analysis, 20 mg of lignin was dissolved in 0.55 mL of anhydrous pyridine/CDCl_3_ (1.6:1, v/v) containing 0.012 mmol of internal standard (endo‐N‐hydroxy‐5‐norbornene‐2,3‐dicarboximide) and 0.0011 mmol of relaxation reagent (Cr(acac)_3_). The mixture was stirred until fully dissolved. Then, 100 μL of 2‐chloro‐4,4,5,5‐tetramethyl‐1,3,2‐dioxaphospholane (Cl‐TMDP) was added to phosphorylate the hydroxyl groups. After 30 min, the sample was transferred to a 5 mm NMR tube and analyzed using the Bruker AVANCE III 500 MHz spectrometer.

### 2.6 Preparation and Characterization of Lignin Nanoparticles

Lignin nanoparticles were prepared using a previously reported self‐assembly method [25, 37]. Due to differences in lignin characteristics, solubility varied among samples; the solvent systems used for each lignin type are detailed in Table S2. After dissolving lignin in the appropriate solvent, a threefold excess volume of water was rapidly added to the lignin solution under mixing to induce nanoparticle formation via precipitation. The organic solvent was allowed to evaporate overnight at room temperature under mixing.

The morphology of the lignin nanoparticles was characterized using FESEM and transmission electron microscopy (TEM). For FESEM analysis, a drop of the lignin nanoparticle suspension was placed on a carbon tape‐coated metal stub and dried at room temperature. The samples were then sputter‐coated with platinum and imaged using a JEOL JSM‐7900F (Japan) microscope at an accelerating voltage of 0.5 kV.

For TEM analysis, lignin nanoparticle suspensions were diluted to a concentration of 0.01 wt%. A drop of 2 wt% uranyl acetate solution was added as a staining agent. The stained suspension was deposited onto a carbon‐coated grid and allowed to stand for a few minutes before excess liquid was removed with filter paper. Imaging was performed using a JEOL JEM‐2200FS (Japan) transmission electron microscope. Diameter of lignin nanoparticles produced from TLA‐lignin was calculated using ImageJ 1.54p software.

## 3 Results

### 3.1 Acid Hydrotropic Fractionation

Plain AHF, performed without any lignin‐modifying additives, served as the reference delignification method. Although previous studies have shown that AHF can nearly completely dissolve lignin from hardwoods such as poplar [33], its efficiency toward softwood has been reported to be significantly lower [34]. This reduced performance is likely due to the more recalcitrant structure of softwood compared to hardwood and nonwoody biomass [38].

In this study, plain AHF treatment of softwood resulted in dark‐colored wood particles. Visual inspection revealed a substantial presence of intact sawdust particles in the delignified samples, indicating that pure AHF does not achieve effective pulping (i.e., the liberation of individual fibers). The yield of the cellulosic fraction was 68.5% (Table 1), and compositional analysis showed that the solid fraction still contained 26.6 wt% lignin (Table 2), suggesting that less than 40% of the original lignin was removed. Most of the yield loss was attributed to the removal of hemicelluloses. The high residual lignin content and the presence of largely undisturbed sawdust particles suggest that plain AHF poorly dissolves the middle lamella, which binds wood tracheids together.

**TABLE 1:** Weighed yield of the cellulosic and lignin fractions based on original wood mass and lignin content in wood and recovery of dissolved lignin by precipitation. Values in parentheses represent factual lignin yield, corrected by subtracting the contribution of chemical modification.

	Cellulosic fraction/wood, %	Yield of lignin/wood, %	Yield of lignin/lignin content in wood, %	Lignin recovery, %
Ref	69	6.3	20	51
EG	72	5.0	16	38
GA	60	14 (13^a^)	42 (41^a^)	70 (68^a^)
Phen	57	25 (19^a^)	79 (59^a^)	96 (72^a^)
ThioU	79	19 (10^a^)	60 (33^a^)	181 (100^a^)
TLA	56	27 (20^a^)	82 (63^a^)	109 (85^a^)

a Chemical modification subtracted.

**TABLE 2:** Chemical composition of solid fraction before and after AHF with lignin‐modifying chemicals and respective removal rates.

	Glucose, %	Xylose, %	Galactose, %	Mannose, %	Acid insoluble lignin, %	Acid soluble lignin, %	Removal, %			
							Lignin	Glucose	Xylose	Mannose
Sawdust	46.9	4.2	1.8	11.0	27.5	4.7	—	—	—	—
Ref	56.9	—a	—a	10.8	23.3	3.3	38.1	21.9	100	45.4
EG	53.4	—a	—a	8.8	25.5	3.6	40.5	12.2	100	29.1
GA	64.2	—a	—a	6.9	17.1	4.4	59.7	17.3	100	62.0
Phen	67.0	—a	—a	9.7	5.1	5.2	81.6	18.1	100	49.6
ThioU	57.2	0.2	—a	11.2	4.1	23.5	32.5	3.5	95.7	19.3
TLA	82.2	1.8	—a	9.6	8.7	5.6	75.3	2.3	76.3	51.4

a Not detected.

Addition of EG into reaction AHF mixture had no notable effect on delignification as the lignin content remained in similar level compared to pure AHF delignification (Table 2). Although EG and other diols have been shown to enhance delignification and yield high‐quality lignin under both acidic [18] and alkaline conditions [39], it is likely that the relatively low dosage used here was insufficient to effectively stabilize lignin under the given conditions.

EG is known to react with lignin—particularly at the β‐O‐4 linkages—forming ether bonds that are more hydrolytically stable than native hydroxy groups [18]. This stabilization can help preserve the lignin's chemical structure. However, in the case of softwood lignin, which naturally contains a higher proportion of carbon–carbon bonds [40], such stabilization may hinder lignin dissolution. If the cleavage of lignin–lignin bonds is suppressed, the already recalcitrant softwood lignin becomes even more resistant to solubilization.

The use of GA as an additive resulted in a notable improvement in delignification, with approximately 60% of the original lignin removed—significantly higher than in plain AHF and EG‐assisted treatments (Table 2). This aligns with previous findings in poplar, where GA‐assisted fractionation yielded a minimal lignin content of 2.8 wt%, compared to 6.4 wt% in plain AHF [35]. Additionally, the yield of isolated lignin in the GA‐assisted system (42%) was double that of plain AHF (20%) (Table 1).

Despite these improvements, the residual lignin content in the softwood sawdust remained above 20 wt%, and only minor fiber liberation was observed during the reaction. Similar to EG, GA is proposed to act as a protective agent that prevents lignin fragmentation [18]. While preserving lignin linkages can be beneficial for downstream applications, reduced fragmentation may hinder delignification of the more recalcitrant softwood lignin. In this context, the stabilization effect of GA may limit the cleavage of lignin–lignin bonds, thereby reducing solubilization efficiency.

Compared to GA, the use of Phen as an additive nearly doubled the lignin yield from 42% to 79% (Table 1), while the residual lignin content in the fibers decreased to 10.3 wt%, significantly lower than in GA‐ and EG‐assisted delignification (Table 2). Among all tested additives, Phen achieved the highest lignin removal efficiency—over 80%.

Previous studies have proposed that phenolic solvents such as thymol [21] and phenol [25] promote lignin dissolution through supramolecular interactions in the presence of acid. However, in the current system, the abundance of competing hydrogen bond donors and acceptors (e.g., water and *p*‐toluenesulfonic acid) likely limits the contribution of such interactions. Instead, it is assumed that Phen acts primarily as a carbocation scavenger, reacting with carbocation centers formed during acid‐catalyzed hydrolysis of lignin [20].

Carbocation formation is a key driver of lignin recondensation, where aromatic units react to form stable carbon–carbon bonds. In this context, low‐molecular‐weight phenol is presumed to react more rapidly with lignin carbocations than larger lignin fragments, thereby suppressing condensation and enhancing lignin solubility.

As a result of the extensive lignin removal, fiber liberation was observed during the reaction with Phen. The resulting cellulosic pulps contained only a few remaining intact sawdust particles, indicating effective delignification and partial pulping.

TLA [30] and ThioU [32] are sulfur‐based nucleophiles presumed to participate in chemical modification of lignin, thereby enhancing its dissolution. Among all tested additives, TLA‐assisted AHF resulted in the highest isolated lignin yield (Table 1). TLA also exhibited the lowest glucose removal rate, with approximately 97% of the original glucose retained in the fibers (Table 2). Notably, TLA was the only additive that preserved a measurable amount of xylose, although nearly 80% was still removed. Similar to Phen, significant fibrillation of sawdust particles was observed, indicating effective fiber liberation.

Although the exact mechanism of TLA‐based delignification is still under investigation, it is hypothesized to follow a pathway similar to thioacidolysis—a method commonly used for lignin structural analysis [31]. The nucleophilic thiol group of TLA reacts with the α‐carbon of lignin, initiating a series of nucleophilic substitutions that ultimately fragment the lignin structure and promote dissolution [41]. Additional reactions, such as thioacetalization of lignin carbonyl groups, may also occur, contributing to the isolation of lignin with low discoloration (see below).

In contrast, ThioU‐assisted AHF resulted in the highest residual lignin content in the fibers (28 wt%, Table 2), which contradicts previous reports of effective softwood delignification using ThioU in aqueous hydrochloric acid at slightly higher temperatures (100°C vs. 80°C used here) [32]. Compared to other systems studied here, ThioU treatment yielded the highest acid‐soluble lignin content (23.5 wt%), nearly five times greater than that of the original wood (Table 2).

The presumed mechanism involves acid‐catalyzed carbocation formation, followed by reaction with ThioU to form isothiouronium groups (i.e., ThioU acts as a carbocation scavenger). In the AHF system, the counterion to the cationic isothiouronium group is the bulky *p*‐toluenesulfonate anion. While this modification does not appear to fragment lignin extensively, the introduction of charged groups likely enhances lignin solubility in aqueous media. However, due to the low water content in AHF, lignin may precipitate upon ethanol addition, resulting in cellulosic fibers with high lignin content. The precipitated cationic lignin is then solubilized in the strong acidic solution used for lignin content analysis, manifesting as a high acid‐soluble lignin fraction.

### 3.2 Properties of Cellulosic Fraction

**FIGURE 2:**
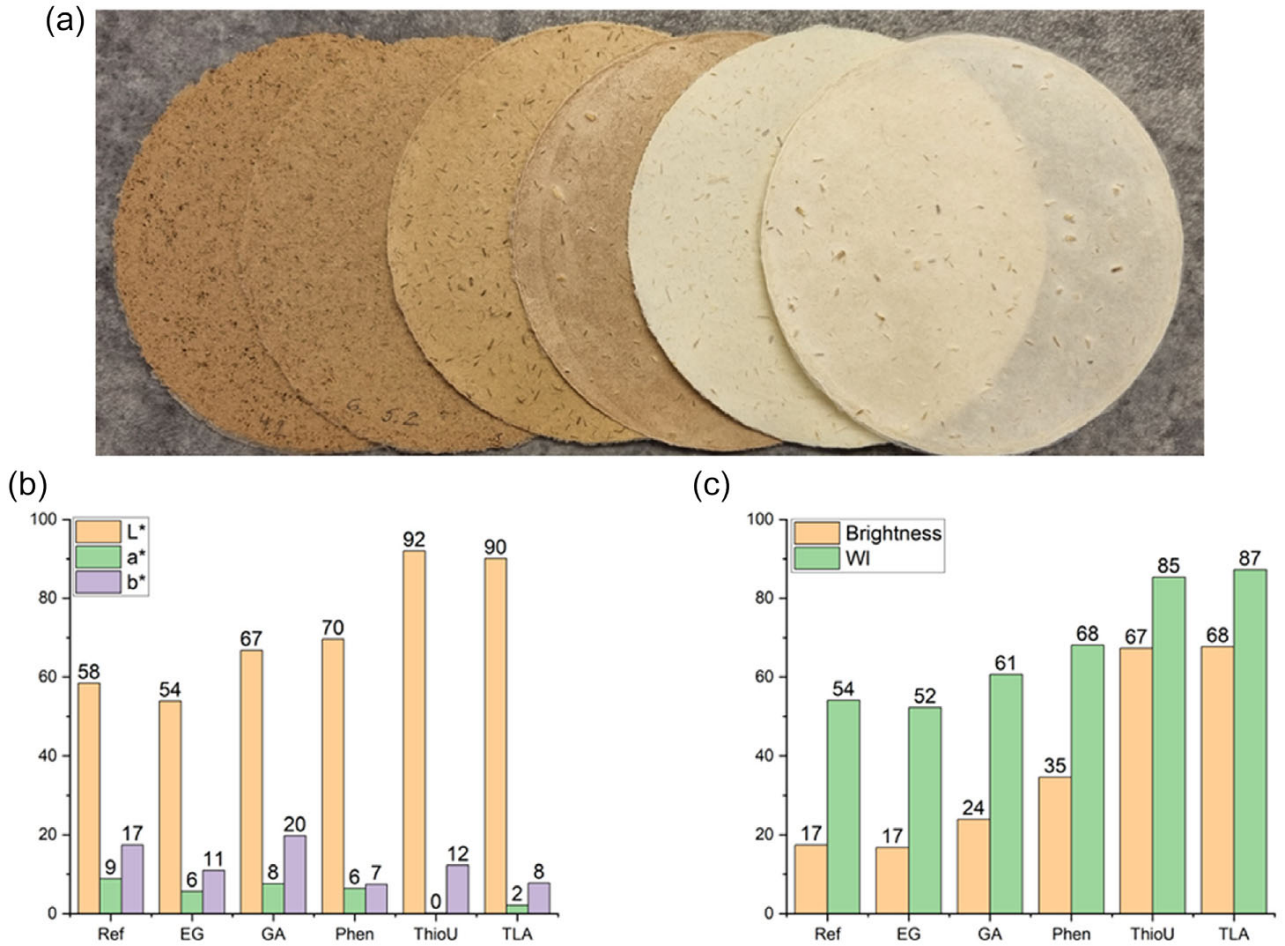
(a) Photograph of sheet produced from delignified softwood (from left to right: Ref, EG, GA, Phen, ThioU, and TLA), (b) *L* ^*^ *a* ^*^ *b* ^*^ color parameters, and (c) brightness and whiteness (WI) of delignified wood.

The color of the cellulosic fractions ranged from brown to whitish (Figure 2a), with corresponding color parameters and ISO brightness values presented in Figure 2b,c. The fractions obtained from Ref‐ and EG‐assisted AHF exhibited the darkest appearance, with whiteness index and ISO brightness values around 50% and 17%, respectively—lower than those typically measured for unbleached kraft pulp [30] and notably lower compared to, for example, formic acid pulping [42].

One of the primary contributors to the dark coloration is the presence of residual lignin, which was relatively high in both Ref‐ and EG‐assisted samples. However, a high lignin content does not necessarily result in dark color, as demonstrated by the ThioU‐treated sample. Despite having the highest residual lignin content (28  wt%), ThioU‐treated fibers exhibited the highest ISO brightness (67%) and whiteness index (85).

This discrepancy suggests that the chemical nature of the residual lignin plays a critical role in color development. In Ref‐ and EG‐assisted AHF, severe lignin condensation and the formation of chromophoric groups likely contribute to the dark appearance. In contrast, ThioU acts as a carbocation scavenger, suppressing lignin condensation. As a result, the residual lignin in ThioU‐treated fibers retains a lighter color.

The color of TLA‐treated fibers was similar to that of ThioU‐treated fibers, further demonstrating that sulfur‐based nucleophilic chemistry is an effective strategy for producing light‐colored cellulosic fractions. Although the color parameters and ISO brightness values of ThioU‐ and TLA‐treated fibers were lower than those reported for thioacidolysis pulping and bleached kraft pulp [30], the mild coloration remains notable when compared to the other treatments studied here. GA‐ and Phen‐treated fibers exhibited intermediate color and brightness values, falling between those of Ref, EG, and the sulfur‐based systems (Figure 2).

The DP of the pulps is presented in Figure S1. While the presence of low‐molecular‐weight compounds such as lignin and hemicellulose may introduce uncertainty in DP calculations, the results provide indicative insights into how different chemistries affect the molecular weight of the resulting cellulosic pulps. Ref‐ and EG‐assisted AHF pulps, which retained high lignin contents, exhibited the lowest DP values (854 and 304, respectively). Interestingly, ThioU‐assisted pulp, despite its similarly high lignin content, showed a higher DP (1157) than Ref and EG pulps. Among all treatments, TLA‐assisted pulp exhibited the highest DP (2257)—approximately 50% greater than the next highest (Phen)—which aligns with previous findings where thioacidolysis pulping yielded high‐molecular‐weight cellulose.

**FIGURE 3:**
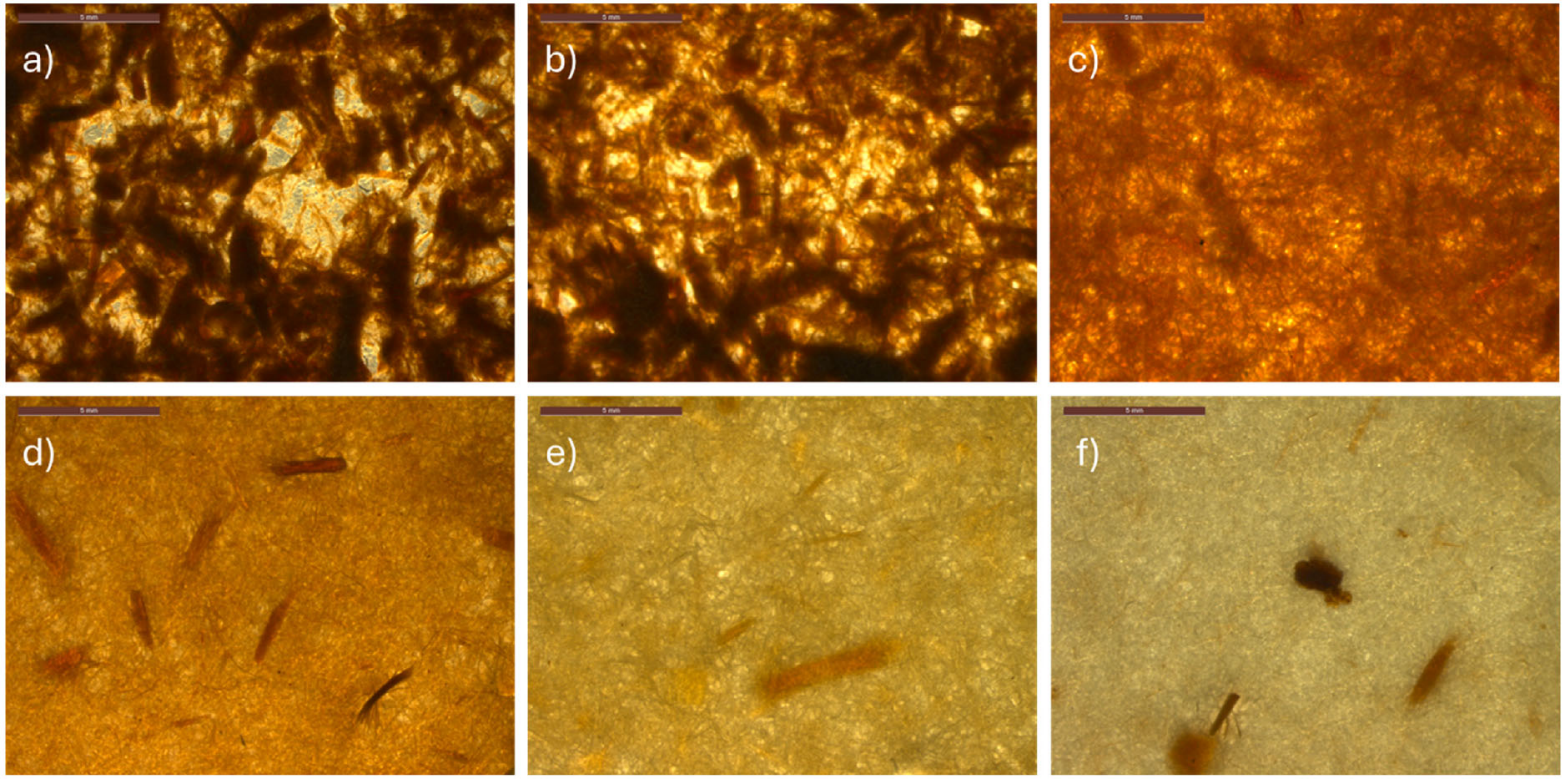
Microscope images of sheets prepared from delignified wood: (a) Ref, (b) EG, (c) GA, (d) Phen, (e) ThioU, and (f) TLA (scale bar: 5 mm).

**FIGURE 4:**
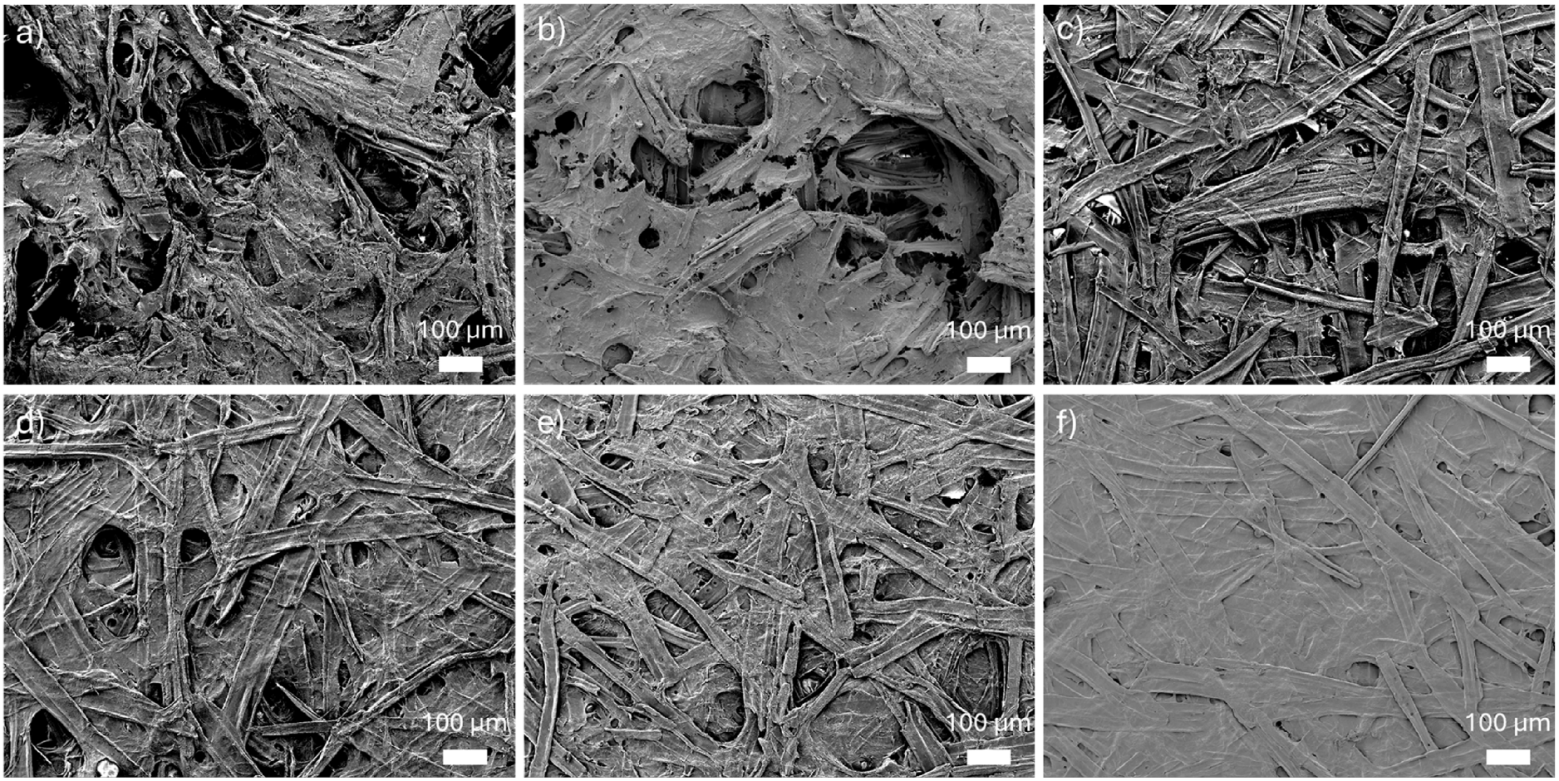
Scanning electron microscope images of sheets prepared from delignified wood: (a) Ref, (b) EG, (c) GA, (d) Phen, (e) ThioU, and (f) TLA.

To evaluate fiber performance, sheets were prepared from the cellulosic fractions and tested for tensile properties (Table 3). Sheets derived from Ref‐ and EG‐assisted AHF exhibited poor mechanical performance, with tensile strengths around 1 MPa. This is consistent with their high lignin content and the presence of nonfibrillated sawdust particles. Without sufficient fiber liberation, a cohesive network cannot form, resulting in low mechanical integrity. Microscopic examination of these sheets (Figure 3a,b) revealed a high proportion of intact sawdust particles, leading to uneven fiber networks with visible voids. These structural defects act as fracture points during tensile testing, contributing to the poor mechanical performance. Similar features—large particles and voids—were also observed in SEM images (Figure 4a,b).

**TABLE 3:** Mechanical properties of sheets prepared from delignified wood.

	Tensile strength, MPa	Tensile modulus, MPa	Strain at maximum tensile force, %	Force at break index, kNm/kg	Tensile stiffness index, MNm/kg	Work capacity, J/kg
Ref	0.78	258	0.46	2.29	0.76	5.28
EG	1.47	450	0.43	3.65	1.12	7.94
GA	11.4	2256	0.63	23.32	4.62	75.87
Phen	28	4178	0.86	44.32	6.68	215.31
ThioU	13.2	1831	0.65	20	5.51	99.14
TLA	31.1	4948	0.78	50.74	8.06	221.22

The GA‐assisted AHF cellulosic fraction exhibited significantly improved mechanical properties compared to Ref‐ and EG‐assisted systems. For example, the tensile strength of GA‐derived sheets was approximately 10 times higher than that of Ref and EG sheets (Table 3). ThioU‐derived sheets showed similar tensile strength (∼13 MPa), despite the notably higher residual lignin content. Visual observations confirmed that ThioU‐assisted delignification led to effective pulping and fiber liberation, which contributed to the formation of a more cohesive fiber network. However, the presence of precipitated lignin on fiber surfaces likely weakened fiber–fiber interactions, limiting mechanical performance.

Morphological differences between GA‐ and ThioU‐derived sheets were evident in light microscopy images (Figure 3c,e). GA sheets contained more non‐disintegrated sawdust particles, although they were notably more homogeneous than those from Ref and EG treatments. ThioU sheets exhibited a relatively compact structure with fewer visible large particles. SEM analysis revealed that ThioU fibers were coated with roundish lignin particles (Figure S2), which may interfere with interfiber bonding, decreasing the mechanical properties of sheet despite the formation of fiber network.

The highest mechanical strength was observed in sheets prepared from Phen‐ and TLA‐treated fibers, both reaching tensile strengths around 30 MPa (Table 3). These samples had similarly low residual lignin contents, which facilitated fiber liberation and the formation of strong interfiber networks. Microscopy images (Figures 3d,f and 4d,f) showed only minor amounts of large particles in both sheets. Notably, the surface of the TLA‐derived sheet appeared particularly smooth, suggesting a high content of fines—small fiber fragments that enhance bonding between larger fibers and reinforce the overall network structure.

### 3.3 Analysis of Lignin

**FIGURE 5:**
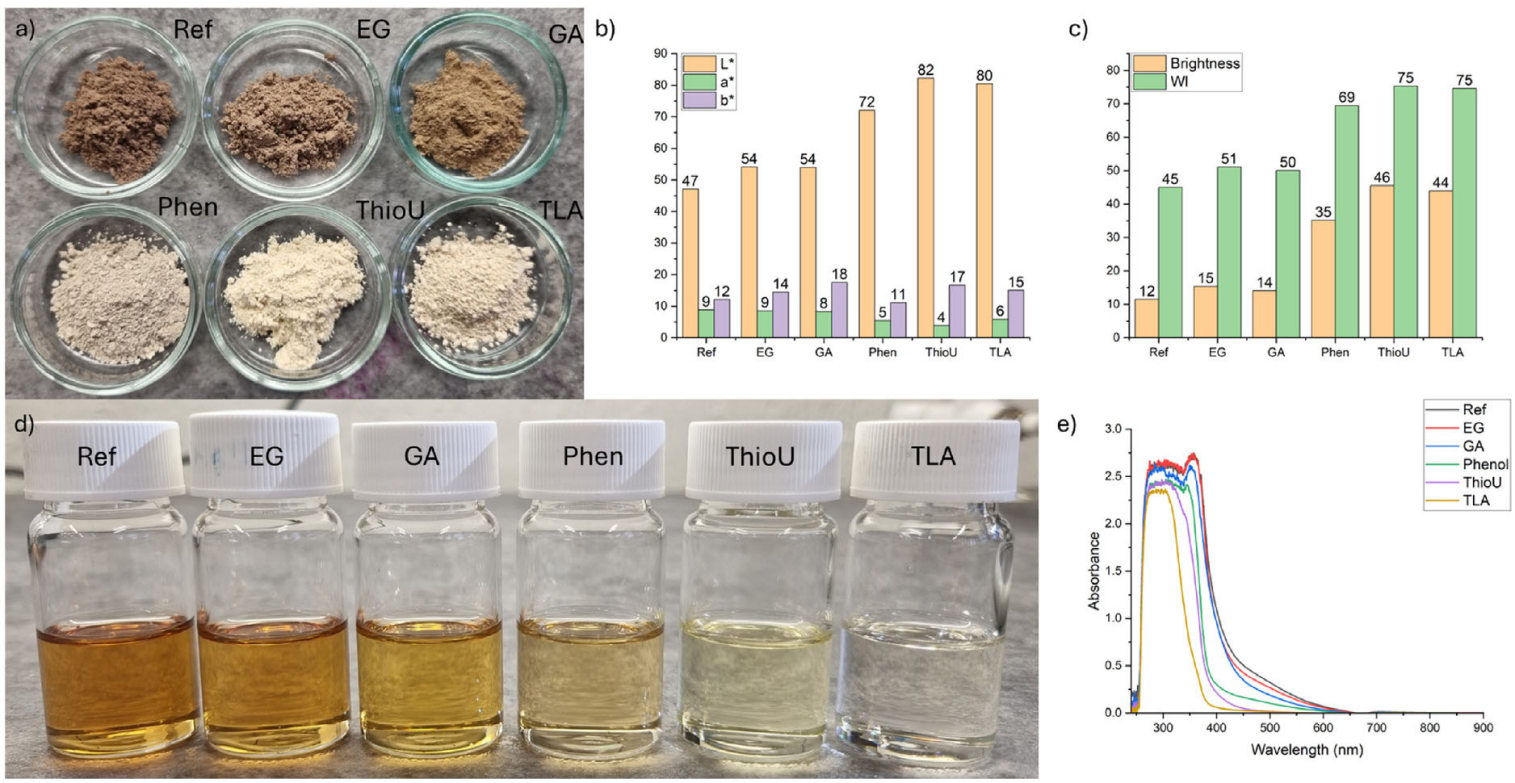
(a) Photograph, (b) *L* ^*^ *a* ^*^ *b* ^*^ color parameters, (c) brightness and whiteness (WI) of isolated lignin, (d) photograph, and (e) UV–Vis spectra of 0.1 wt% lignin solutions in dimethyl sulfoxide.

Photographs of lignin samples obtained via AHF with various additives are shown in Figure 5a. The color of the lignin ranged from dark brown to off‐white. The darkest fractions, with the lowest color values, were produced using Ref, EG‐, and GA‐assisted AHF methods (Figure 5b,c). In contrast, Phen‐lignin exhibited a relatively light color (L* value of 72) with a noticeable reddish hue. Previously, an L* value of 86 was reported for a similar system using aqueous *p*‐toluenesulfonic acid and Phen with pine as the raw material [43]. The difference may be attributed to variations in process conditions, including a higher Phen concentration (23.1 vs. 7.6 wt%), lower acid concentration (46.9 vs. 72  wt%), and shorter reaction time and lower temperature (15 min at 70°C).

Consistent with observations from the cellulosic pulps, ThioU‐ and TLA‐assisted AHF produced the lightest lignin fractions (Figure 5a–c). Although their color values were lower than those of white lignin obtained via thioacidolysis pulping [30], they are comparable to—or even exceed—those reported for most lignin samples in the literature [44, 45, 46, 47, 48]. These results further support the conclusion that sulfur‐based nucleophiles are highly effective in producing mildly colored lignocellulosic materials, likely due to their ability to suppress lignin condensation.

Similar trends in lignin color observed in the solid state were also evident in solution. When dissolved in DMSO (0.1 wt%), lignin from Ref, EG‐, and GA‐assisted AHF appeared the most brownish, while TLA‐lignin produced an almost colorless solution (Figure 5d). UV–Vis spectra revealed that all lignin samples exhibited strong absorption in the UV region. However, TLA‐lignin showed absorption primarily in the UVB range (280–315 nm), whereas the other lignins also absorbed in the UVA region (315–400 nm) (Figure 5e).

These differences suggest potential application‐specific advantages. ThioU‐ and Phen‐lignin may be suitable for applications requiring broad‐spectrum UV protection, such as sunscreens [49]. In contrast, TLA‐lignin, with its selective UVB absorption, could be advantageous in agricultural applications like plant cultivation, where UVB filtering is beneficial [50, 51]. While Ref‐, EG‐, and GA‐lignins also offer broad UV‐blocking properties, their strong visible light absorption would result in significant coloration—undesirable in many applications. However, in some contexts, such as sunscreens, visible light absorption may be beneficial for pigmentation protection [52].

**FIGURE 6:**
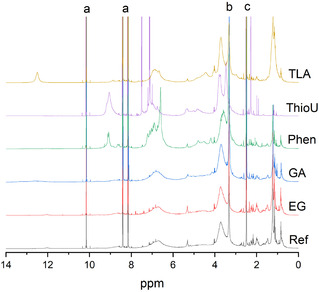
^1^H NMR spectra of lignin samples (strong peaks in the spectra are related to a: 4‐nitrobenzaldehyde used as internal standard, b: water, and c: DMSO).

^1^H NMR spectra of Ref‐, EG‐, and GA‐lignins exhibited similar features, including signals from aliphatic protons (0.5–2.5 ppm), a strong methoxy peak at 3.7 ppm, and aromatic protons in the 6–8 ppm range (Figure 6). A broad, low‐intensity peak around 12.5 ppm was observed in the GA‐lignin spectrum, which may originate from carboxylic acid groups introduced into the lignin structure during fractionation in the presence of GA.

Phen‐lignin exhibited distinct features in the aromatic region of the ^1^H NMR spectrum compared to Ref‐, EG‐, and GA‐lignins. A strong peak at 6.6 ppm and four additional peaks between 7.2 and 7.9 ppm were observed. The 6.6 ppm signal is likely attributable to the ortho‐position proton of a phenolic group introduced at the α‐position of the β‐O‐4 unit [20]. The presence of multiple new aromatic peaks suggests that Phen is incorporated into the lignin structure via both ortho‐ and para‐substitution, consistent with the strong directing effect of the hydroxy group. Additionally, an unknown peak at 9.1 ppm was detected, potentially corresponding to aldehyde groups, although this assignment remains tentative.

In the ThioU‐lignin spectrum, sharp peaks at 2.3, 7.1, and 7.5 ppm were observed, which can be attributed to the methyl and aromatic protons of the *p*‐toluenesulfonate group—serving as the counterion to the isothiouronium group. A signal at 9.1 ppm corresponds to protons of the isothiouronium group itself [53]. These findings support the proposed mechanism of lignin modification during AHF in the presence of ThioU.

TLA‐lignin displayed a prominent peak at 12.5 ppm, indicative of carboxylic acid functionality, along with a strong aliphatic signal centered at 1.2 ppm. These features strongly suggest the incorporation of the TLA moiety into the lignin structure.

To further investigate lignin modification during fractionation, the content of hydroxy and carboxylic acid groups was quantified (Table 4). Ref‐ and EG‐lignins exhibited similar functional group profiles, consistent with their comparable delignification performance. In contrast, Phen‐lignin showed a markedly elevated content of *p*‐hydroxyphenyl groups (1.7 mmol/g), more than tenfold higher than in other samples. This increase is a clear indication of lignin phenolation during AHF in the presence of Phen and aligns well with previous reports [43].

**TABLE 4:** Quantification of aliphatic and phenolic hydroxy and methoxy (OMe) groups of isolated lignin samples.

	Aliphatic, mmol/g	Phenolic, mmol/g					OMe, mmol/g
		C5‐substituted	Guaiacyl	p‐Hydroxy‐phenyl	Total phenolic	Carboxylic acid	
Ref	1.56	0.86	0.68	0.13	1.67	0.32	2.11
EG	1.72	0.82	0.70	0.12	1.64	0.28	3.3
GA	1.52	0.8	0.38	0.10	1.28	0.60	3.43
Phen	1.72	0.70	0.63	1.70	3.03	0.18	2.71
ThioU	—a	—a	—a	—a	—a	—a	1.57
TLA	1.14	0.66	0.46	0.07	1.19	1.46	2.77

a Not determined.

Both GA‐ and TLA‐lignin showed notable presence of carboxylic acid. In case of GA‐lignin, carboxylic acid content was 0.6 mmol/g, being around twice the amount of carboxylic acid in Ref‐lignin. The carboxylic acid content of TLA‐lignin was 1.5 mmol/g, indicating higher reactivity of TLA with lignin compared to GA. The presence of a notable amount of carboxylic acid is in line with the proposed reaction product obtained using GA and TLA during the AHF (Figure 1).

Due to poor solubility of ThioU‐lignin in deuterated pyridine, ^31^P NMR analysis of hydroxy and carboxylic acid groups could not be performed. However, elemental analysis revealed sulfur and nitrogen contents of 1.91 and 1.78 mmol/g, respectively. These values are in good agreement with the proposed structure of ThioU‐modified lignin, where ThioU is covalently attached via formation of an isothiouronium group, with *p*‐toluenesulfonate as the counterion (Figure 1). The slight excess of sulfur may be attributed to partial hydrolysis of the isothiouronium group to a thiol [54].

The main lignin interunit linkages identified by HSQC NMR are summarized in Table 5 (HSQC spectra are presented in Figure S3). Ref‐, EG‐, and GA‐lignins exhibited relatively high β‐O‐4 content compared to the other samples, with GA‐lignin showing the highest value. These samples also had the highest levels of β–β and β–5 linkages, indicating a more condensed lignin structure due to the presence of C—C bonds.

**TABLE 5:** Interlinkage units of isolated lignin samples.

	Interlinkage units (per 100 aromatic group)				
	β‐O‐4/α‐OH	β‐β_α_	β‐β_β_	β‐5_α_	β‐5_β_
Ref	1.8	1.71	6.56	2.54	5.71
EG	1.96	2.39	7.81	4.17	11.4
GA	3.63	4.16	14.03	4.33	6.81
Phen	0	0.53	1.32	0.12	1.19
ThioU	0.39	0.33	0.55	0.89	1
TLA	0.92	1.57	1.99	1.23	0.93

In contrast, Phen‐lignin showed no detectable β‐O‐4 linkages, while ThioU‐ and TLA‐lignins had β‐O‐4 contents below 1 per 100 aromatic units. This suggests that the use of these additives during AHF leads to near or complete conversion of β‐O‐4 linkages. Notably, Phen‐, ThioU‐ and TLA‐lignins also exhibited lower β–β and β–5 contents compared to Ref‐, EG‐, and GA‐lignins. This indicates that the absence of β‐O‐4 linkages is not due to self‐condensation, but rather to chemical modification of lignin by highly nucleophilic additives.

### 3.4 Lignin Nanoparticles

Lignin nanoparticles represent an emerging class of nanomaterials [55], utilized in diverse applications ranging from composites [56] to cosmetic [57], such as sunscreens [58]. In this study, lignin samples were employed in nanoparticle production via solvent–antisolvent‐induced self‐assembly methods [37]. Due to their distinct chemical characteristics, the lignin samples exhibited varied solubility profiles and could not be dissolved in a single universal solvent. The solvents used for each lignin sample are listed in Table S2.

All lignin samples yielded stable nanoparticle dispersions. The color of each dispersion corresponded closely to the color of the lignin in its solid state (Figure S4). TLA‐ and ThioU‐lignin dispersions appeared white to the naked eye, while Ref‐, EG‐, and GA‐lignin samples produced distinctly brown dispersions. Phen‐lignin resulted in a pink dispersion.

**FIGURE 7:**
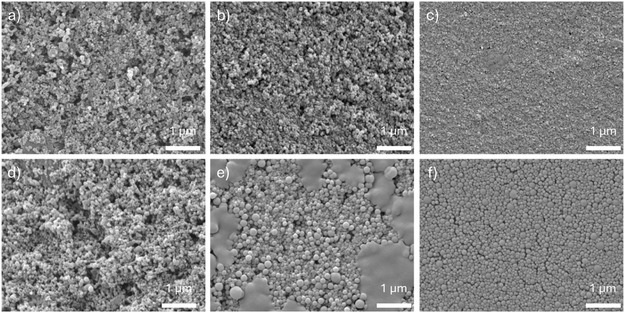
SEM images of lignin nanoparticles produced from lignin isolated by (a) Ref, (b) EG, (c) GA, (d) Phen, (e) ThioU, (f) TLA.

**FIGURE 8:**
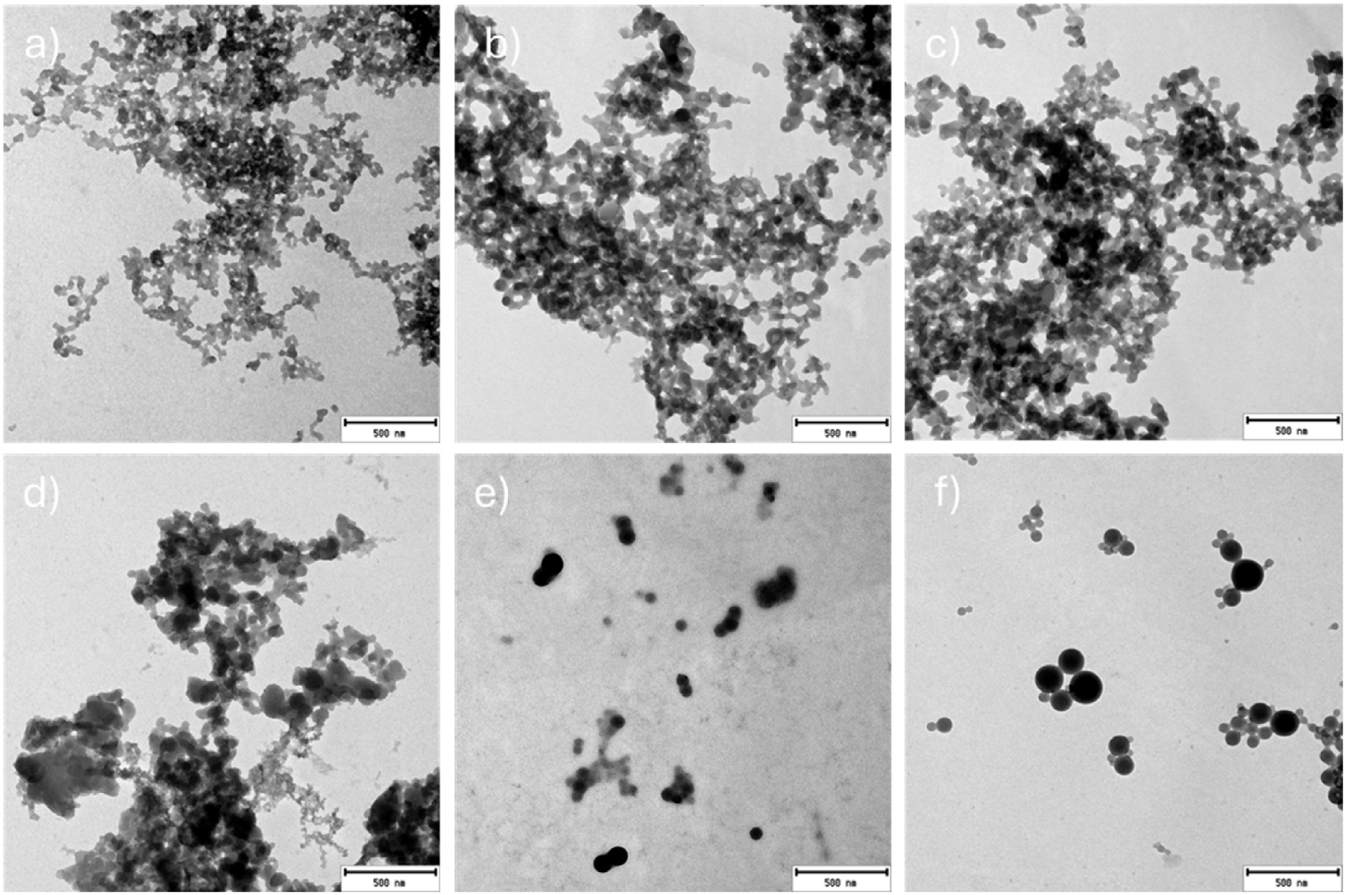
TEM images of lignin nanoparticles produced from lignin isolated by (a) Ref, (b) EG, (c) GA, (d) Phen, (e) ThioU, (f) TLA.

SEM imaging (Figure 7) revealed that TLA‐lignin formed spherical lignin nanoparticles with diameters ranging from tens to hundreds of nanometers (Figure 7f). This spherical morphology was confirmed by TEM imaging (Figure 8f), and the particle diameter was calculated to be 68 ± 36 nm (see histogram in Figure S5). Similar spherical nanoparticles were observed for ThioU‐lignin (Figure 7e); however, these were partially covered by a film‐like material, suggesting that water addition did not result in complete lignin precipitation. The polyelectrolyte nature of ThioU‐lignin, attributed to the presence of isothiouronium groups, renders it at least partially water‐soluble, leading to incomplete precipitation upon water addition. The nonprecipitated lignin subsequently forms film‐like sheets atop the lignin nanoparticles.

Phen‐, GA‐, EG‐, and Ref‐lignin formed very small particles, with sizes ranging from tens to hundreds of nanometers (Figure 7a–d). However, TEM images showed that most of these lignin particles lacked well‐defined structures and appeared fused together, forming irregularly shaped aggregates (Figure 8a–d). While TLA‐nanoparticles also exhibited some degree of aggregation in TEM images, distinct boundaries between individual particles were still visible (Figure S6a). In contrast, for example, EG‐nanoparticles did not display such clear separation lines (Figure S6b), indicating that they not formed by fusion between individual nanoparticles, but exhibited irregular shapes. Similar irregularly shaped nanoparticles have previously been reported for lignin isolated using both alkaline and acidic DES [59].

## 4 Discussion

As noted above, softwood is a challenging biomass to delignify, as demonstrated here by the low delignification efficiency of pure AHF under conditions previously reported to enable near‐complete delignification of hardwood [33]. In addition to the limited delignification, significant darkening of the cellulosic fraction was observed. This darkening is likely due to lignin condensation and the formation of chromophoric groups, such as conjugated carbonyls.

Although EG has been shown to be an effective solvent and additive for biomass delignification, it did not yield a notable improvement compared to pure AHF. The effect of EG is attributed to the nucleophilic properties of its hydroxy groups, which can react with carbocations formed during acid‐catalyzed lignin degradation. This mechanism is comparable to that of TLA, where the thiol (–SH) group acts as a nucleophile. However, a clear difference in delignification efficiency was observed between EG‐ and TLA‐assisted fractionation, likely due to the higher nucleophilicity of thiols compared to alcohols. The more nucleophilic SH group in TLA reacts more readily with lignin intermediates than the OH group in EG. Additionally, other factors—such as the cleavage of interunit linkages in lignin—may contribute to the observed differences. According to the proposed mechanism of lignin thioacidolysis, multiple thiol groups can react with lignin structures (e.g., β‐O‐4 linkages), ultimately leading to lignin fragmentation. In contrast, alcohols like EG primarily react with the α‐carbon of β‐O‐4 linkages, forming more stable ether bonds that hinder further fragmentation. Furthermore, higher reactivity of TLA compared to EG efficiently suppress the condensation of lignin, diminishing the redeposit of lignin on the fiber surface.

Unlike TLA, it is presumed that the use of ThioU do not lead to the cleave lignin structures. Instead, its effect is based on the formation of cationic isothiouronium groups, which increase lignin's hydrophilicity and thus its solubility in water. However, without molecular fragmentation, high‐molecular‐weight lignin remains only partially soluble and is precipitated during washing. This solubility limitation could be mitigated by using an ethanol–water mixture during the washing step.

Phen behaves similarly to EG, ThioU, and TLA in that it acts as a carbocation scavenger. While phenolation of kraft lignin has been proposed to induce bond cleavage (e.g., β‐O‐4) [60], model compound studies with syringol suggest that selectively arylates the α‐carbon of β‐O‐4 linkages during the biomass fractionation [20]. Unlike ThioU, the introduction of phenolic groups may enhance lignin solubility in ethanolic washing liquors due to the introduction of phenolic hydroxy group. Compared to EG, ThioU, and TLA, Phen is aromatic and can diffuse more effectively into lignin‐rich regions during fractionation. This facilitates rapid reaction with lignin intermediates, suppressing self‐condensation and resulting in high delignification efficiency.

In contrast to the other chemicals studied, GA does not act as a carbocation scavenger but instead prevents carbocation formation via acetalization. Based on this mechanism, GA might be expected to perform similarly to Phen, TLA, and ThioU. However, it exhibited significantly lower delignification efficiency. One possible explanation is insufficient acetal bond formation, allowing carbocation formation and subsequent lignin condensation. Furthermore, although GA‐assisted delignification has been successful with other biomass types, the lower efficiency observed here may also be due to the specific chemical structure of softwood. In all lignocellulosic species, lignin is covalently linked to carbohydrates—mainly hemicellulose—via ether and ester bonds. Softwood lignin, which contains more carbon–carbon bonds than other lignin types, also has a relatively high content of benzyl ether linkages to carbohydrates via the α‐carbon [61]. Acid‐catalyzed cleavage of these benzyl ether bonds can generate carbocations, which, in the case of GA‐assisted fractionation, may lead to lignin condensation due to the lack of nucleophilic properties of GA. Although the AHF reaction mixture contains an excess of aromatic p‐toluenesulfonic acid, which could theoretically act as a carbocation scavenger, the strong electron‐withdrawing nature of the sulfonic acid group reduces the nucleophilicity of the aromatic ring, limiting its effectiveness in this role [62]. It is also noteworthy be mentioned, that although chemical, such as EG, which have previously been used efficiently in lignin preservation did not work in this study, no optimization was performed here, and different conditions (e.g., with higher chemical dosage) should be studied in future to get attain more knowledge on these chemicals.

## 5 Conclusions

Five different functional chemicals were investigated for their ability to chemically modify lignin during AHF of softwood lignocellulose. Sulfur‐containing nucleophiles were found to react readily with lignin during fractionation, resulting in the lightest‐colored cellulosic fibers and lignin fractions. This indicates that TLA and ThioU effectively suppress the formation of chromophoric groups in lignin. TLA yielded high lignin removal efficiency and produced fiber sheets with good mechanical properties. In contrast, ThioU led to lower lignin yield due to precipitation during washing, and the resulting fiber sheets exhibited lower mechanical properties, likely due to lignin coverage on the fiber surface. Phen achieved the highest lignin removal efficiency, although the color of both the cellulosic and lignin fractions was slightly darker compared to sulfur‐based chemicals. Compared to reference AHF without additives, GA significantly improved fractionation efficiency, though its effect was less pronounced than that of Phen and the sulfur nucleophiles. EG showed no notable impact. Nanoparticle analysis revealed that Ref‐, EG‐, GA‐, and Phen‐lignin samples formed small, irregularly shaped nanoparticles, while ThioU‐ and TLA‐lignin produced more uniform, spherical nanoparticles. However, ThioU‐lignin nanoparticles were partially covered with nonprecipitated lignin. Overall, the results demonstrate that chemical modification of lignin during fractionation can significantly enhance process efficiency. However, chemicals that are highly effective for delignifying hardwood or nonwood biomass may not perform as well with more recalcitrant feedstocks like softwood, highlighting the need for tailored approaches in lignocellulose processing.

## Supporting Information

Additional supporting information can be found online in the Supporting Information section.

## Funding

This work was supported by Business Finland (10894/31/2022 and 14/31/2023).

## Conflicts of Interest

The authors declare no conflicts of interest.

## Supporting information

Supplementary Material

## Data Availability

The data that support the findings of this study are available from the corresponding author upon reasonable request.
