# Supplementary material for: Comparative Evaluation of Additives in Softwood Fractionation: Impacts on Lignin Recovery and Pulp Quality
Source: ChemSusChem. 2026 Apr 18;19(8):e70640. doi: 10.1002/cssc.70640 (PMC13091184; doi:10.1002/cssc.70640)
Supplement: Supplementary file 1 — Supplementary Material [file CSSC-19-e70640-s001.pdf]

Comparative Evaluation of Additives in Softwood Fractionation: Impacts on Lignin Recovery and Pulp Quality

Juho Antti Sirviö,[1]* Ekaterine Sheridan,[2] Donya Arjmandi,[3] Jasmiina Haverinen,[4] Dmitry Tarasov,[3] Chunlin Xu,[3] Ari Ämmälä,[1] Jarkko Räty[4]

[1]Fibre and Particle Engineering Research Unit, University of Oulu, P.O. Box 4300, 90014 Oulu, Finland E-mail: juho.sirvio@oulu.fi

[2]Max Planck Institut für Kolloid- und Grenzflächenforschung Am Mühlenberg 1, 14476 Potsdam, Germany

[3]Laboratory of Natural Materials Technology, Åbo Akademi University, Turku, 20500, Finland

[4]Kajaani University Consortium, Measurement Technology Unit, University of Oulu, Kehräämöntie 7, Kajaani, 87400, Finland

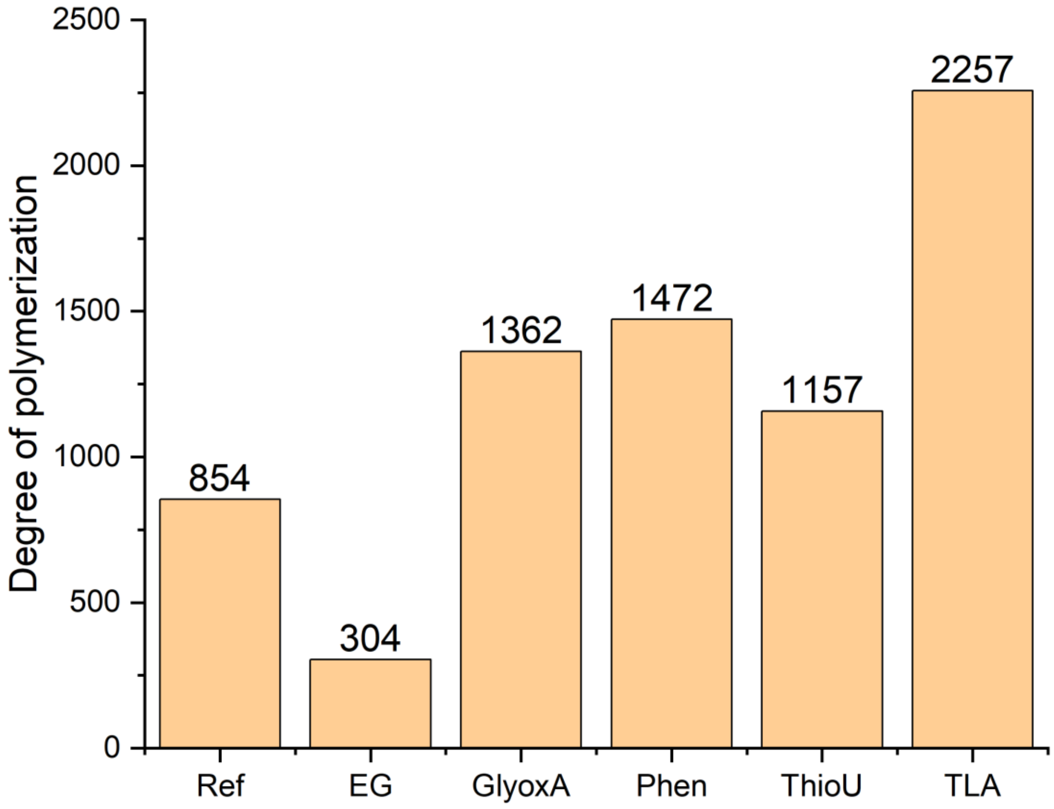

Figure S1. Degree of polymerization of cellulosic fraction.

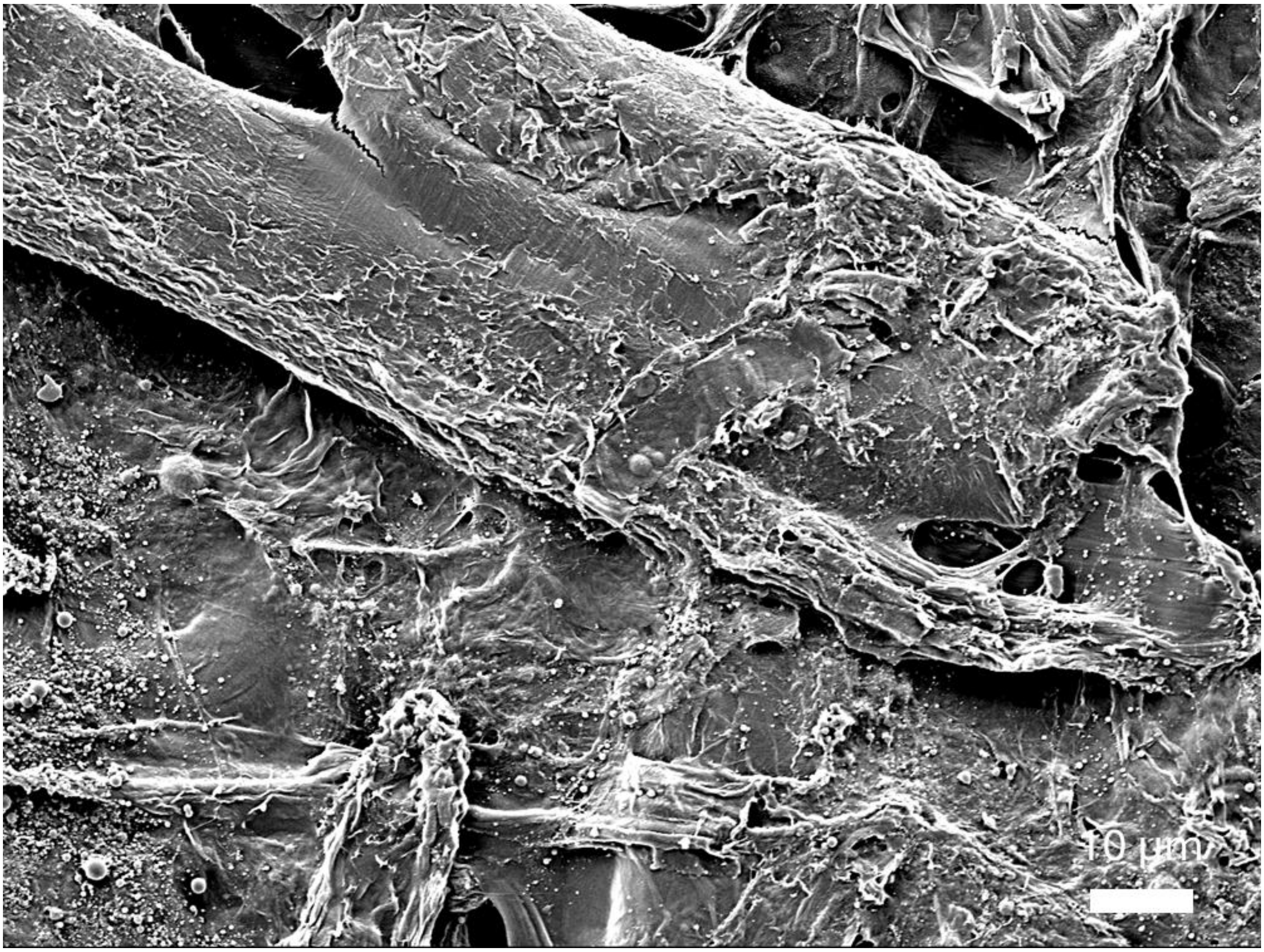

Figure S2. SEM image of the ThioU-fiber sheet showing precipitated lignin particles.

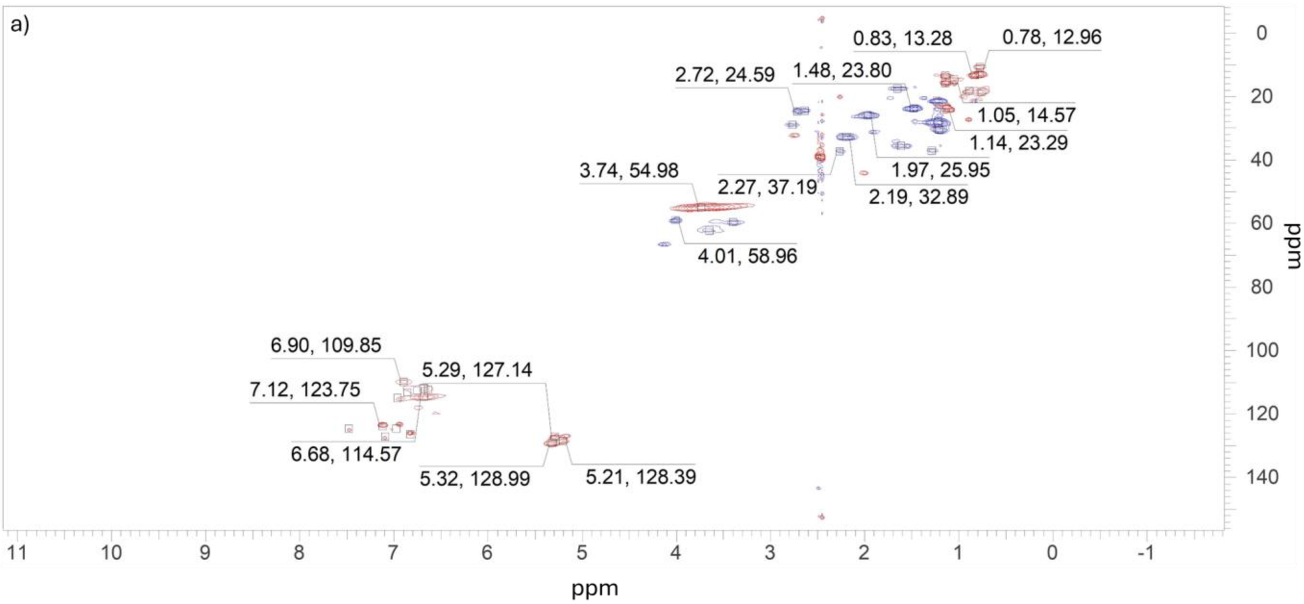

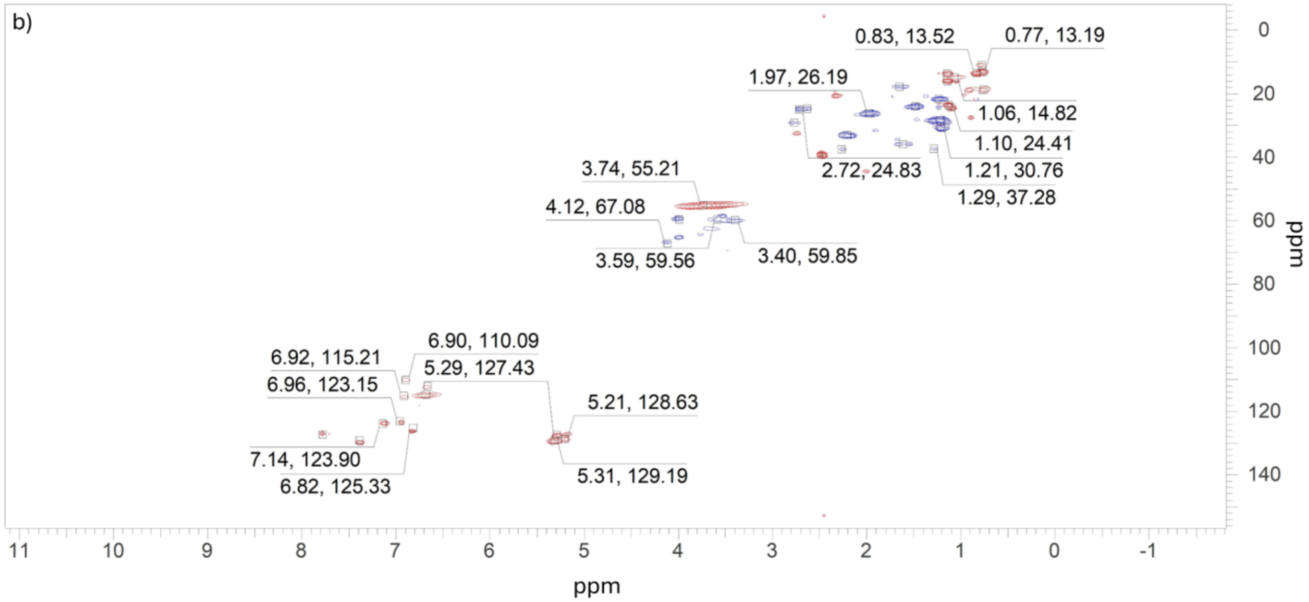
b)
0.83, 13.52
0.77, 13.19
1.97, 26.19
1.06, 14.82
1.10, 24.41
1.21, 30.76
1.29, 37.28
2.72, 24.83
3.74, 55.21
4.12, 67.08
3.59, 59.56
3.40, 59.85
6.92, 115.21
6.90, 110.09
5.29, 127.43
6.96, 123.15
5.21, 128.63
7.14, 123.90
6.82, 125.33
5.31, 129.19
ppm
ppm

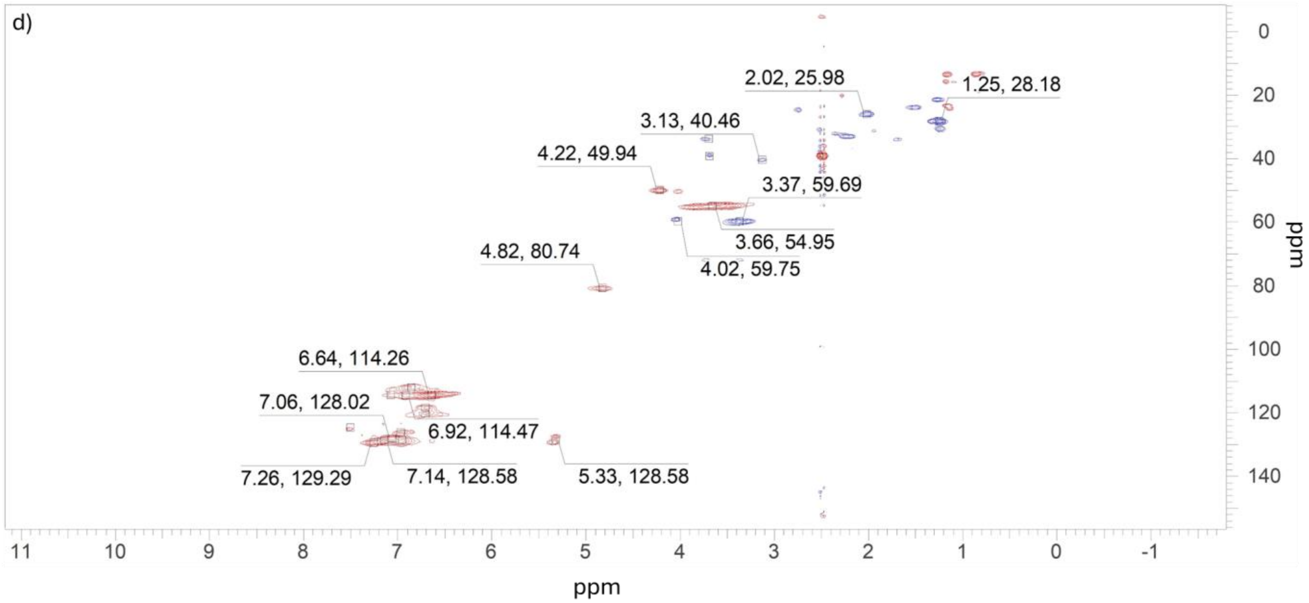
d)
2.02, 25.98
1.25, 28.18
3.13, 40.46
4.22, 49.94
3.37, 59.69
3.66, 54.95
4.02, 59.75
4.82, 80.74
6.64, 114.26
7.06, 128.02
6.92, 114.47
7.26, 129.29
7.14, 128.58
5.33, 128.58
ppm
ppm

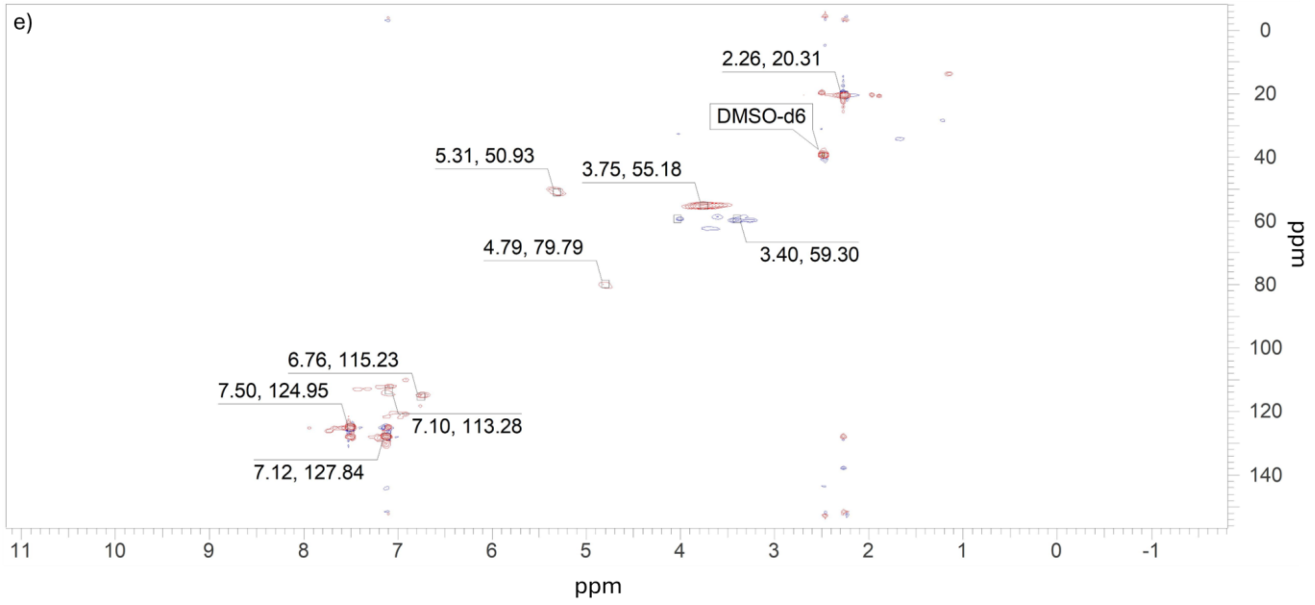

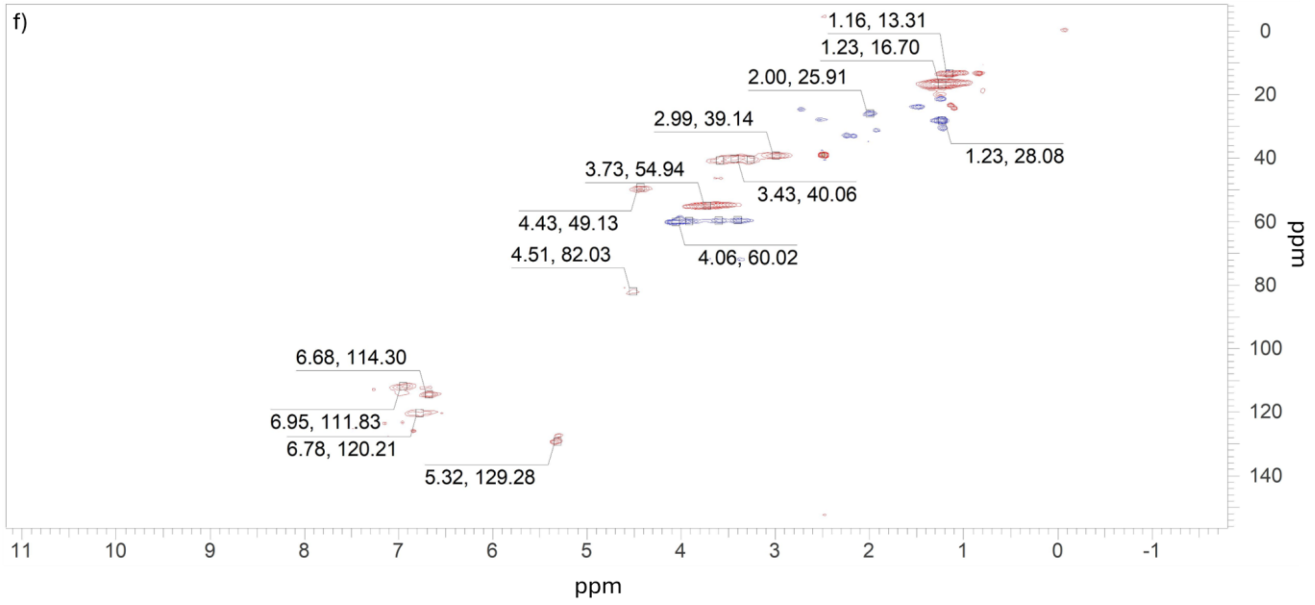

Figure S3. HSQC spectra of lignin samples: a) Ref, b) EG, c) GA, d) Phen, e) ThioU, and f) TLA.

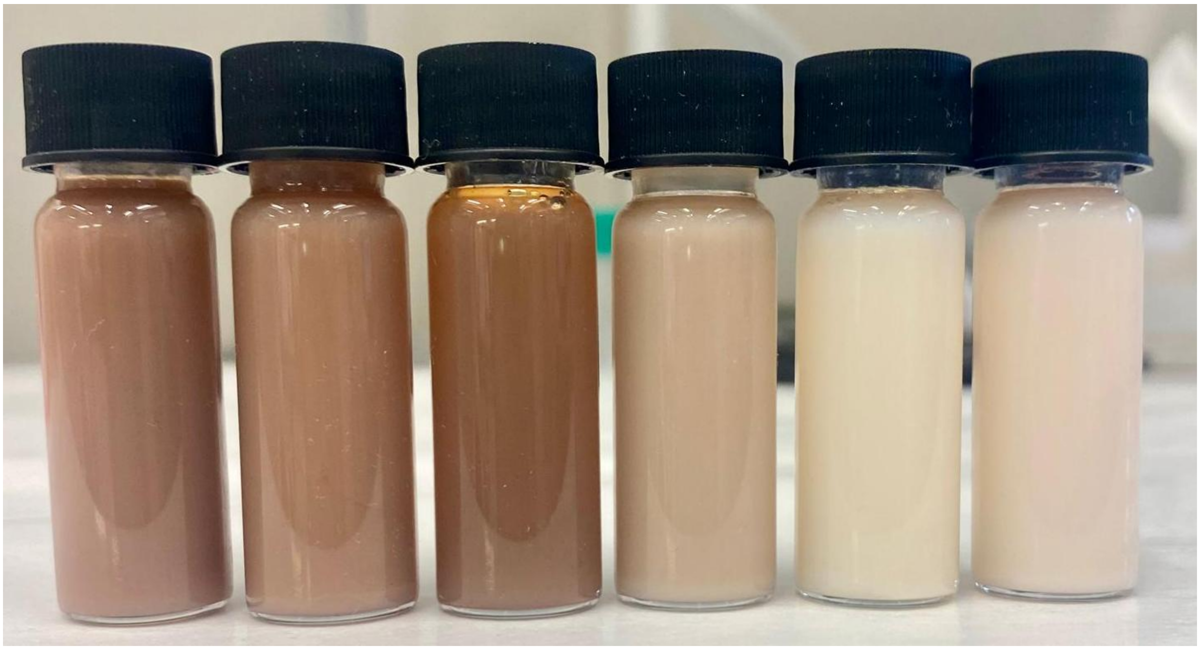

Figure S4. Photograph of lignin nanoparticle suspensions from left to right: Ref, EG, GA, Phen, ThioU, and TLA.

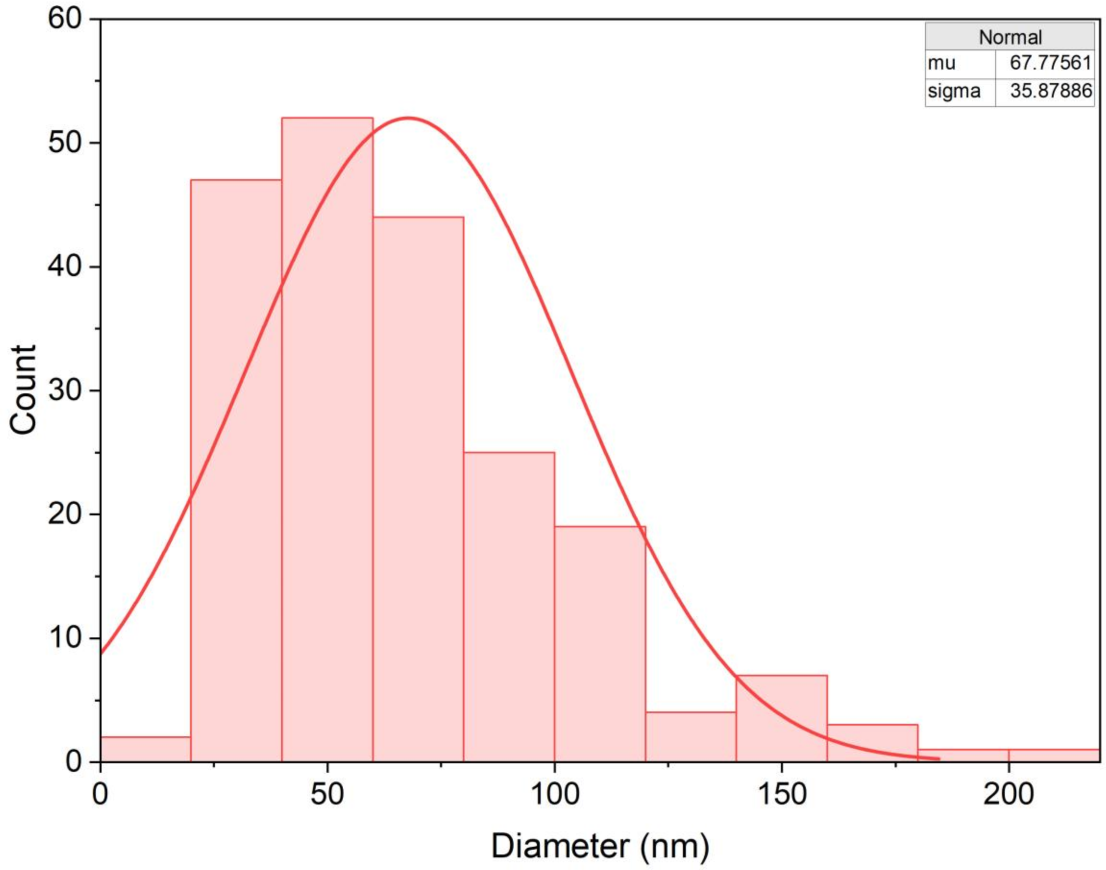

Figure S5. Histogram of the diameter of lignin nanoparticles produced from TLA-lignin.

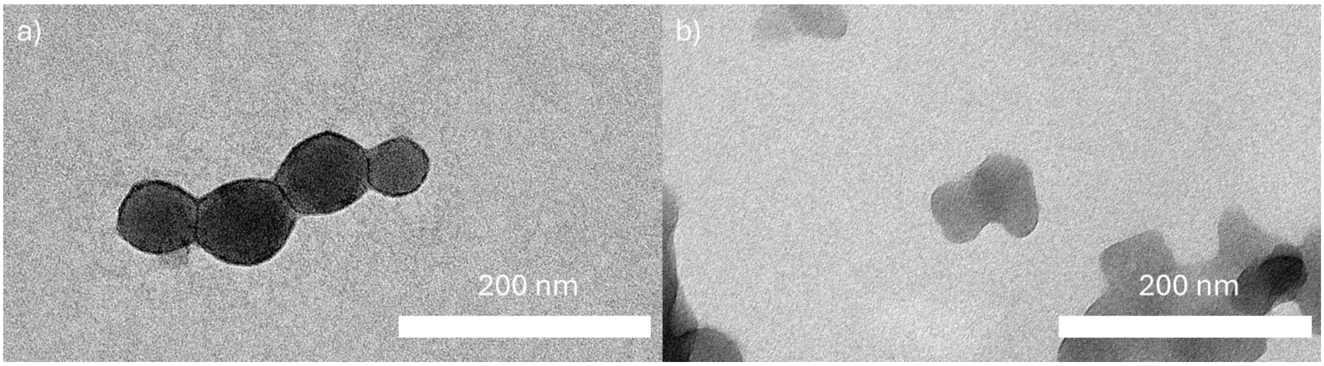

Figure S6. TEM image of lignin nanoparticles obtained using a) TLA and b) EG as chemical additive during AHF.

Table S1. Mass fraction (%) of chemicals used in plain AHF and with chemical additives.

Sample	p-toluenesulfonic acid monohydrate	Water	Ethylene glycol	Glyoxylic acid monohydrate	Phenol	Thiourea	Thiolactic acid
Ref	80	20	-	-	-	-	-
EG	80	14.97	5.03	-	-	-	-
GA	80	12.54	-	7.46	-	-	-
Phen	80	12.4	-	-	7.6	-	-
ThioU	80	13.8	-	-	-	6.2	-
TLA	80	11.4	-	-	-	-	8.6

Table S2. Solvent used in preparation of lignin nanoparticles. Mass fractions of mixed solvent presented in parenthesis.

Sample	Solvent
Ref	Acetone
EG	Tetrahydrofuran
GA	Acetone:Water:Ethanol (1:1:1)
Phen	Acetone:Water:Ethanol (3:1:1)

ThioU	Acetone:Water (1:1)
TLA	Acetone
